# An Account of Some Extraordinary Symptoms Which Were Apparently Connected with Certain Morbid Alterations about the Veins and Nerves

**Published:** 1795

**Authors:** John Pearson

**Affiliations:** Surgeon of the Lock Hospital, and of the Public Dispensary.


V. *An Account of ſome extraordinary Symptoms which were apparently connected with certain morbid Alterations about the Veins and Nerves. Communicated in a Letter to Dr.* Simmons *by Mr.* John Pearſon, *Surgeon of the Lock Hoſpital, and of the Public Diſpenſary.*

MRS. P. aged fifty-one years, of Miles' Lane, Cannon Street, began to ſuffer from a peculiar uneaſineſs at the inner part of her left leg, about ſeventeen years ago, when ſhe was in the third month of her ſecond pregnancy. The ſkin which covered the particular ſeat of her complaint, retained its natural colour; but there was a circular induration, of about half an inch in diameter, very little elevated above the ſurface, which was exquiſitely painful when ſlightly touched or compreſſed; this morbid part was ſituated in the courſe of the vena ſaphena major, and was about ſix inches above the joint of the ancle. Beſides the acute pain which was produced by inadvertently touching this little tumour, Mrs. P. commonly ſuffered ſeveral paroxyſms of of pain every day; each of thefe attacks was accompanied with an increafed rednefs, and a fenfible elevation of the indurated part, the pain at the fame time extending to the knee, and often darting to the ftomach; the duration of the fit was about twenty minutes; it was attended with flight convulfive motions of different parts of the body, and frequently terminated with flatulent eructations. Thefe fits of pain did not recur at any regular periods; fo that the number which fhe underwent in the courfe of a day was various and uncertain; for a difordered ftate of the ftomach, or a fudden perturbation of mind would at any time excite one of the paroxyfms. She alfo had obferved, that the feverity of her fufferings was invariably increafed during the periods of menftruation and of pregnancy; and that in the latter months of geftation, the duration of each recurrence of pain was extended to an hour and a half. But although this difeafe was uniformly aggravated by certain alterations in the ftate of the uterus, yet it continued with undiminifhed feverity after Mrs. P. had ceafed to bear children; for when her youngeft child was more than fix years old, fhe had not experienced any abatement of her daily fufferings. About thirteen years ago, I advifed her to have the morbid part removed; but at that time fhe was unwilling to undergo an operation; fhe however fubmitted to various methods of treatment, under the direction of different medical gentlemen, but without obtaining any relief.

In the month of April, 1793, Dr. Lowder, who had been long acquainted with the circumftances of this painful complaint, informed Mrs. P. of the fuccefs which had attended the removal of a fimilar tumour, by the application of a cauftic. She read the hiftory of the cafe, which is publifhed in the third volume of the Memoirs of the Medical Society of London, and very foon determined to feek relief from the fame mode of treatment.

Accordingly, on the 22d of April, I applied the lapis infernalis to the morbid part; fhe endured the moft excruciating tortures during feveral minutes after its application; but the pain gradually diminifhed with the fenfibility of the part, fo that in about twenty minutes the efchar was completely formed, and fhe then felt no more pain than what is the ufual confequence of a cauftic applied to any part of the body. From this day fhe never experienced the recurrence of a fingle paroxyfm of pain; the efchar exfoliated in about twelve days; and on the 7th of June the fore was perfectly healed.

As the preceding hiftory contains fome curious and rather uncommon circumftances, I beg leave to offer a few obfervations upon fome of them. The indurated part having been deftroyed by a cauftic, it was not in my power to examine its internal ftructure, fo as to difcover the true nature of the morbid alteration. I afcertained, however, that a portion of the vena faphena major, and that branch of the crural nerve which accompanies it in its courfe down the infide of the leg, were completely included within this tumour. This fact was clearly demonftrated after the exfoliation of the efchar; for I then faw a portion of the vein hanging down at the fuperior part of the fore, and the naked nerve in contact with it; and on touching the nerve with my probe, Mrs. P. inftantly complained of an acutely painful fenfation, fimilar to that which fhe had been accuftomed to feel before the tumour was removed. I then deftroyed that part of the nerve which was expofed with lunar cauftic, and my patient fuffered no more uneafinefs. After thus proving that a vein, and a confiderable ramification of a nerve, were contained within the difeafed part, I proceed to obferve, that the paroxyfms of pain were excited by every thing that accelerated or otherwife difturbed the circulation of the blood; whether applied to the induration, or affecting the general fyftem; as all ftrong exertions of the mufcles, external impulfe, or mental commotion. The afcent of the blood, in the veins of the lower extremities, is neceffarily impeded in the ftate of pregnancy; and during this period, the fits of pain were always fharper, and were alfo of longer duration; and at the time of parturition, when the action of the heart and blood-veffels is confiderably increafed, Mrs. P. fuffered exceedingly; for, to ufe her own expreffion, fhe "had all her labour pains in her leg."

It is alfo highly probable, that the portion of vein which paffed through the tumour was unufually diftended with blood at the time of the paroxyfm; for upon thefe occafions, the morbid furface became redder than common; and the tumour was fenfibly elevated. We may therefore, perhaps, venture to conclude, that the vein and the nerve being confined within a fubftance that could not be eafily diftended, whenever ever the vein became preternaturally turgid, the nerve was compreſſed between its parietes and the internal ſurface of the induration; and that conſequently the ſymptoms were connected with this ſtate of the part. I do not ſuppoſe that it will be neceſſary for me to undertake a proof in detail, that a certain degree of preſſure upon a nerve will produce pain, ſpaſms, and convulſions; it may be ſufficient for my purpoſe to refer to a few of the many inſtances which are recorded in medical books. In the fourth volume of the Edinburgh Medical Eſſays, Dr. Short has related the hiſtory of an epilepſy, which was cauſed by the preſſure of a hard cartilaginous ſubſtance upon a nerve; he cured his patient by removing the tumour, and dividing the nerve. Guattani, in his Treatiſe *de externis Aneuryſmatibus*, (Hiſt. XX.) has recorded a caſe in which violent ſpaſms were occaſioned by the preſſure of an aneuriſm upon a nerve. In the Eſſays and Obſervations Phyſical and Literary, Vol. III., the late Sir John Pringle has publiſhed a Caſe, where a tumour formed by extravaſated blood, by preſſing upon the intercoſtal nerves, produced pain, irritation, and perhaps a hiccup, which could not be ftopped*. I do not intend to deduce any general conclufion from a particular inftance; for although the remarkable fymptoms which occurred in Mrs. P.'s cafe, were connected with a morbid ftate of a vein and a nerve; yet as no account has been publifhed of the internal ftructure of parts which have been affected by a fimilar complaint, it would be improper to conclude, that every inftance of local morbid fenfibility, accompanied with convulfive motions and pain, muft depend upon fuch a peculiar condition of the fuffering parts. I have indeed feen another cafe, very much refembling that of Mrs. P.'s, in which there is a fmall exquifitely fenfible induration

* For inftances of convulfive motions, and even epilepfy, produced by local difeafes about fome of the extremities, or that were cured by the removal of matter, carious bone, or fome portion of the integuments, confult Willis *de Morbis Convulf.*; Riverius *de Epilepfia*; Schenckii *Obfervat.* (Lib. *de Epilepfia.*); Foreftus *de Cerebri Morbis*, Lib. X. Obf. 67; Petri Borelli *Hiftor. & Obfervat. medico phyficarum*, Cent. II. Obf. 95. Joh. Rhodii *Obferv. Med.* Cent. I.; Tulpii *Obferv. Med.* Lib. IV. Cap. 2; Boneti *Sepulchretum*, Lib. I. Sect. 13; Van Swieten *Comment. in Aph.* H. Boerhaave, Tom. III. § 1075. Haller *Elementa Phyfiologiæ*, Tom. IV. § 30. Simfon on the Vital and Animal Actions, Effay I. ch. 3.

at at the pofterior part of the leg, near the beginning of the tendo achillis, from which the patient fuffers acutely whenever it is touched. She has occafional paroxyfms of pain, but they return at uncertain intervals; and fhe thinks that they grow milder. In this inftance, as in that recorded by Dr. Biffet *, the tumour becomes uneafy in rainy and windy weather; but it does not appear that the difeafe had ever any connexion with pregnancy. I fufpect that the tumour, which I have juft now mentioned, may be connected with the vena faphena minor, and that confequently it may include or comprefs a fmall branch of the fciatic nerve; but as I could not render the cutaneous veins of the leg turgid by moderate preffure, its exact fituation was not afcertained †.

In

* Memoirs of the Medical Society of London, Vol. III. Art. VI.

† The firft volume of M. Pouteau's pofthumous works contains a very curious hiftory of a difeafe which he there calls cancerous; whether properly or no I fhall not inquire; but as it refembles Mrs. P.'s cafe in fome of its characters, I fhall take the liberty of prefenting an abftract of it:

" On voyoit à la partie baffe du *Sternum* une furface " ovale de largeur d'un ecu de fix livres dans fon petit dia-

 " metre,

In the early part of the laſt Spring, a young married woman applied to me at the Public Diſpenſary, complaining of pain and lameneſs of the right arm. She ſhewed me a tumour of a pale red colour, and of about the ſize of a filberd,

" metre, ſans elévation, ſans rougeur, ſans engorgement " circonvoiſin. La peau ſeulement qui la recouvroit étoit " un peu moins nette, que par tout ailleurs, mais ſemblable " à la ſenſitive qui paroit craindre la main qui l'approche. " Cette portion des tégumens auroit fait reſſentir les plus " vives douleurs, ſi le doigt, ſans la toucher, en eût ap- " proché avec trop de célérité. Le moindre inſećte, un " fetu que le hafard auroit fait poſer deſſus, euſſent auſſi- " tôt rappellé les convulſions. Les rétours de ces convul- " ſions étoient periodiques, ſe montrant à ſept heures & " demie préciſes du ſoir. Dans le plus grand calme, on ne " les attendoit que de deux jours l'un; & à la moindre agita- " tion, les mouvemens convulſifs étoient journaliers. Leur " durée étoit de deux heures, & même plus." The hiſtory preſents us with many other extraordinary circumſtances; but it may be ſufficient at this time to add, that M. Pouteau made a crucial inciſion in this morbidly ſenſible part, which afforded an immediate although but a temporary ſuſpenſion of the pain and convulſions. He then extirpated the portion of diſeaſed integuments; but as the young lady was not perfećtly relieved by this operation, he finally completed the cure by burning a cylinder of cotton upon the part. Vide *Oeuvres poſthumes de M. Pouteau*, Tom. I. ch. 1.

which which was ſituated in the courſe of the vena mediana baſilica, at the bend of the arm: this morbid part was conſtantly uneaſy; but when it was preſſed or handled, ſhe complained of acute pain, which extended along the upper arm, and produced ſlight convulſive motions in the muſcles. She derived no advantage from mild diſcutient and emollient applications; but her pain increaſed ſo much, that her health became injured, and ſhe was at length confined to her bed. On viſiting her at home, I found the tumour unaltered in its appearance, excepting a ſpontaneous ſeparation of the cuticle from its ſurface; ſhe was in conſtant pain; the uneaſineſs not only proceeding along the upper arm, but alſo to the neck, and affecting the breaſt and muſcles on the right ſide. Her pulſe was feeble, but not too frequent; ſhe complained of a great ſenſe of weakneſs, and convulſive motions were excited in the muſcles of the upper arm, neck, and thorax, on that ſide, by the gentleſt examination of the morbid part. I ordered a large veſicatory to be applied on the inner part of the fore arm, and directed her to take ten grains of *pulvis ipecacuanhæ compoſitus*, whenever her pain ſhould be unuſually ſevere. She ſoon derived conſiderable relief from this mode of of treatment: the bliftering plafter was repeated twice during my attendance; the tumour gradually became lefs painful, and diminifhed in bulk; and in about a month it had entirely difappeared. It was not more than three weeks after fhe was difmiffed, when fhe applied to me again, on account of a tumour very much refembling the former one, which was fituated at the bend of the arm, in the courfe of the vena cephalica; fo that a portion of the vein evidently paffed through, or, rather, was included within the center of the morbid part. The pain and morbid irritability affected the fame parts as before, but in a much inferior degree. I directed a mode of treatment fimilar to that which had been employed on the former occafion, and it was attended with equal fuccefs.

This young woman had fome fymptoms which indicated a difeafed ftate of the lungs; and fhe occafionally fpat blood: but fhe had not been formerly fubject to any particular complaints; fhe menftruated regularly; and had never been pregnant. I cannot affign any probable caufe for the appearance of fo fingular a complaint as that which I have now defcribed; but fome of the effects which took place would perhaps perhaps admit of an explanation, if it could be proved that a ſmall ramification of a nerve, as well as a portion of a vein, were included within each of the tumours. That this was actually the caſe is highly probable, becauſe the cutaneous nerve diſtributes ſeveral of its branches in the vicinity of the vena mediana baſilica; and ſmall fibrils belonging to the muſculo-cutaneous nerve, are commonly ſeen near the vena cephalica, and the vena mediana cephalica; ſo that tumours ſituated at the bend of the arm, and in the courſe of theſe blood veſſels, muſt be almoſt neceſſarily in contact with one or more branches belonging to the internal, or external cutaneous nerves *.

* The late Profeſſor Camper, in a valuable work entitled *Demonſtrationum Anatomico-Pathologicarum Liber primus continens Brachii humani Fabricam et Morbos*, has given a very diſtinct view of the mode in which theſe ſmall branches of nerves are diſtributed at the bend of the arm; and his engravings are accompanied with ſome good practical obſervations. Mr. Abernethy alſo publiſhed two engravings, laſt year, in the ſecond part of his Surgical and Phyſiological Eſſays, in which the courſe of theſe nerves is very neatly and correctly delineated: and the eſſay to which they are annexed, contains many uſeful remarks "on the ill conſequences ſometimes ſucceeding to venæ-"ſection."

I beg

I beg leave to refer it to the intelligent reader, how far the following account of a difeafe of the fubcutaneous nerves, as defcribed by Profeffor Camper in the work already referred to, bears any refemblance to the preceding hiftories.

"Non raro in nervis cutaneis tubercula par-
"va ac dura obfervantur, quæ vera ganglia
"funt, pifi magnitudinem licet non excedant;
"dies tamen noctefque acutiffimis lancinanti-
"bus doloribus ægros torquent: externis re-
"mediis non cedunt; fcalpello igitur ea attin-
"gere oportet. Franequeræ ex cubito feminæ
"tale, plagâ factâ, fuftuli, quod ramo mufculo-
"cutanei nervi adhærebat: poft operationem
"optime fe habuit. In fubcutaneis nervis fre-
"quenter effe videntur. Amftelædami fimile
"ganglium genu mulieris occupans, eodem
"modo fanari curavi. In viris plus femel ea
"vidi: albicant intus, cartilagineæ duritiæ
"funt, renitentia, & intra nervorum tunicas
"fedem habent." Lib. I. P. 11. Cap. 2. § 5.

I have feen many fymptoms refembling thofe which occurred in the preceding cafes, apparently follow, as confequences of wounds inflicted on fmall branches of nerves; but as this paper is already much longer than I expected it would have been, I muft defer giving an account of them to another opportunity. As the following cafe exhibits fome uncommon circumftances, I infert it as a kind of fupplement to the foregoing hiftories.

" The fingular effects of an iffue in the in-
" fide of the thigh, which appeared in the cafe
" of a clergyman; written by himfelf, Auguft
" 25th, 1793.

" The Rev. Dr. T——, of Knightfbridge,
" above 60 years of age, having had a hint
" from a medical friend, that an iffue might be
" of ufe to his health, he had one made by a
" blifter, in the lower part and at the infide
" of his right thigh, about the end of May laft.
" Two days after the pea was put in, he was
" feized with a ficknefs and vomiting, which
" continued feveral hours. In about fix days
" after this firft attack, he had a return of the
" fame fymptoms; and thefe fits recurred every
" fix or feven days. But what is very remark-
" able, when the iffue began to difcharge, he
" became deaf in both his ears, and the deaf-
" nefs arrived to fuch a degree, that in preach-
" ing he could but juft hear his own voice.

" After the iffue had been kept open fix
" weeks, it occurred to him, that perhaps the
" regular fits of ficknefs and vomiting, and the

" unufual

" unufual deafnefs, (both of which he recol-
" lected had commenced with the iffue) were
" occafioned by a fympathy of the nerves; and
" having made obfervations for one week longer,
" which confirmed this opinion, he determined
" to dry it up. This he did gradually, by ufing
" peafe of a fmaller fize, till the ulcer was not
" more than one eighth of an inch in diameter.
" When the pea had been out only twelve hours,
" he was fenfible of fome fmall return of his
" hearing, and on looking at the fore, he found
" it healed; which he confidered as a farther
" confirmation of his opinion, refpecting the
" caufe of his deafnefs, as well as of the ficknefs
" and vomiting. He found, that as the wound
" healed, the deafnefs leffened, and when it was
" completely healed, his hearing was quite reco-
" vered, nor has he had one fit of ficknefs fince."

When Dr. T—— related his cafe to me, I defired him to let me fee the cicatrix of the iffue; and on carefully examining it, it appeared probable that the pea had preffed againft the fide of the vena faphena. I would alfo farther add, that my examination of the part excited a flight degree of naufea.

